# Time-dependent progress of lower urinary tract dysfunction in streptozotocin-induced diabetic rats with or without low-dose insulin treatment

**DOI:** 10.7150/ijms.95461

**Published:** 2024-04-29

**Authors:** Nailong Cao, Rong Lv, Daisuke Gotoh, Eduardo C. Alexandre, Naoki Yoshimura, Baojun Gu

**Affiliations:** 1 Department of Urology, Shanghai Sixth People's Hospital Affiliated to Shanghai Jiao Tong University School of Medicine, Shanghai, China.; 2 Department of Urology, University of Pittsburgh School of Medicine, Pittsburgh, Pennsylvania, USA.

**Keywords:** Diabetes Mellitus, Lower Urinary Tract Dysfunction, Insulin

## Abstract

**Objectives:** To examine time-dependent functional and structural changes of the lower urinary tract in streptozotocin-induced diabetic rats with or without low-dose insulin treatment and explore the pathophysiological characteristics of insulin therapy on lower urinary tract dysfunction (LUTD) caused by diabetes mellitus (DM).

**Methods:** Female Sprague-Dawley rats were divided into five groups: normal control (NC) group, 4 weeks insulin-treated DM (4-DI) group, 4 weeks DM (4-DM) group, 8 weeks insulin-treated DM (8-DI) group and 8 weeks DM (8-DM) group. DM was initially induced by i.p. injection of streptozotocin (65 mg/kg), and then the DI groups received subcutaneous implantation of insulin pellets under the mid dorsal skin. Voiding behavior was evaluated in metabolic cages. The function of bladder and urethra *in vivo* were evaluated by simultaneous recordings of the cystometrogram and urethral perfusion pressure (UPP) under urethane anesthesia. The function of bladder and urethra *in vitro* were tested by organ bath techniques. The morphologic changes of the bladder and urethra were investigated using Hematoxylin-Eosin and Masson's staining.

**Results:** Both 4-and 8-weeks diabetic rats have altered micturition patterns, including increased 12-h urine volume, urinary frequency/12 hours and voided volume. *In-vivo* urodynamics showed the EUS bursting activity duration is longer in 4-DM group and shorter in 8-DM group compared to NC group. UPP change in 8-DM were significantly lower than NC group. While none of these changes were found between DI and NC groups. Organ bath showed the response to Carbachol and EFS in bladder smooth muscle per tissue weights was decreased significantly in 4- and 8-weeks DM groups compared with insulin-treated DM or NC groups. In contrast, the contraction of urethral muscle and maximum urethral muscle contraction per gram of the tissue to EFS stimulation were significantly increased in 4- and 8-weeks DM groups. The thickness of bladder smooth muscle was time-dependently increased, but the thickness of the urethral muscle had no difference.

**Conclusions:** DM-induced LUTD is characterized by time-dependent functional and structural remodeling in the bladder and urethra, which shows the hypertrophy of the bladder smooth muscle, reduced urethral smooth muscle relaxation and EUS dysfunction. Low-dose insulin can protect against diuresis-induced bladder over-distention, preserve urethral relaxation and protect EUS bursting activity, which would be helpful to study the slow-onset, time-dependent progress of DM-induced LUTD.

## Introduction

A healthy lower urinary tract (LUT) function relies on the coordination between bladder and urethra, which is regulated by the central and peripheral nervous system 1. Due to the intricate neural regulatory mechanisms and urinary continence-maintaining mechanism, the LUT is susceptible to a wide range of disorders, including diabetes mellitus (DM) 2, 3. DM-induced LUT dysfunction (LUTD) has been extensively documented, including diabetic urethral dysfunction, which is in most cases overlooked in comparison to diabetic bladder dysfunction 4, 5. DM may initially promote urethral dysfunction characterized by increased urethral pressure during voiding, which may be attributed to the impairment of the urethra smooth muscle (USM) relaxation mechanism 4. As the disease progresses, it can cause impairment of coordinated micturition due to dyssynergic activity of the external urethra sphincter, resulting in detrusor-sphincter dyssynergia (DSD) 5. Therefore, similar to diabetic bladder dysfunction 6, the time-dependent changes of urethral function in DM are also existed and need further exploration and elucidation 7.

Although streptozotocin (STZ)-induced diabetic animal model has been widely used in previous studies 8, 9. It is usually a short-term model, with diabetes symptoms peaking within a few weeks after induction. This contrasts with the chronic nature of diabetes in humans and does not fully replicate the pathological and physiological processes of human diabetes. Therefore, we employed low-dose insulin approximately 2 units per 24 hours) treatment to maintain relatively stable glucose levels, slightly elevated within the range of 200 and 300 mg/dL (median level), and established a new animal model. Furthermore, we found that urethral function could be protected after insulin treatment compared to STZ-induced DM animal model, and it may be ascribed to inhibit the damage of nitric oxide (NO) pathway after insulin treatment. However, the time-dependent changes of the bladder and urethra have not been established in insulin-treated DM animal model.

Therefore, in this study, we established an insulin-treated DM animal model, analyzed the time-dependent functional and structural changes in the bladder and urethra, and then clarified the pathophysiological mechanism in DM and insulin-treated DM animal models.

## Materials and Methods

### Animal model establishment

Eighty-five adult female Spraque-Dawley rats (Hilltop Laboratory, Pittsburgh, Pennsylvania) weighing between 250 to 300 gm, were included in the study. DM was induced by a single intraperitoneal injection of STZ (65 mg/kg) freshly dissolved in sodium citrate buffer. Blood glucose levels were monitored weekly using a blood glucose meter (Freestyle Lite, USA). Rats were considered to have DM when blood glucose reached or exceeded 300 mg/dL. Age-matched controls received a vehicle and rats with induced DM received subcutaneous implantation of LinBit insulin pellets (LinShin Canada, Ontario, Canada) under the mid dorsal skin. The release rate of insulin in the body was about 2 units per 24 hours. The aim was to maintain blood glucose levels between 200 and 300 mg/dL in insulin-treated DM rats. Female Sprague-Dawley rats were divided into five groups: normal control (NC) group, 4-week insulin-treated DM (4-DI) group, 4-week DM (4-DM) group, 8-week insulin-treated DM (8-DI) group and 8-week DM (8-DM) group. All animal experiments were conducted in accordance with the ARRIVE and NIH guidelines and approved by the Institutional Animal Care and Use Committees (IACUC) (Protocol approval #18090279). Efforts were made to minimize the suffering and the number of animals needed to obtain reliable results.

### Metabolic cage study

Twenty-five of the rats (n = 5 per group) were housed in metabolic cages to assess their voiding behavior over a 12-hour period during the nocturnal phase (7PM to 7AM, lights off). Subsequently, all rats underwent cystometry and EUS electromyography (EUS-EMG) tests.

### Cystometry and EUS electromyography with open urethra

The rats that underwent metabolic cage study (n=5 per group) were anesthetized with urethane (1.0 g/kg, i.p.). Bladder and urethra were exposed via a lower midline abdominal incision. a catheter (PE-50 Smiths Medical) was inserted through the top of the bladder to measure the intravesical pressure (Pves). The bladder catheter was connected to a pressure transducer and an infusion pump for cystometrogram (CMG) assessment. Physiological saline at room temperature was infused in the bladder to elicit repeat voiding responses. The infusion rate was 0.1 mL/min for the different groups. The following parameters were measured: bladder capacity, voided volume (VV), voiding efficiency (VE), inter-contraction interval (ICI), bladder compliance, maximum of Pves (Pves_max_), EUS activity duration and EUS bursting activity duration. VV was determined by subtracting the post-void residual from the calculated bladder capacity. Bladder compliance was calculated according to the following formula: compliance = bladder capacity / (pressure at volume threshold for inducing a voiding contraction minus initial pressure at the start of saline infusion). VE was determined by the MV/bladder capacity x100. These parameters were evaluated using a PowerLab unit and LabChart (AD Instruments, Colorado Springs, CO, USA).

To record EUS-EMG, two fine insulated silver wire electrodes (0.05-mm diameter) with exposed tips were inserted into lateral sides of the mid-urethra, targeting muscle fibers of the EUS. EUS-EMG was recorded and analyzed using PowerLab unit and Labchart (AD Instruments). EUS-EMG data were sampled at 5,000 Hz, 5-10 times the frequency of the major peaks as recommended to prevent aliasing. After the test finished, the bladder and urethra were extracted for Haematoxylin & Eosin (H&E) and Masson's staining.

### *In vivo* functional studies with simultaneous recordings of intravesical pressure under isovolumetric conditions and urethral perfusion pressure (UPP)

The separate group of rats (n=6 per group) were anesthetized with isoflurane and urethane (1.0 g/kg, i.p.), and the bladder and ureters were exposed via a lower midline abdominal incision. The bladder neck was tied to allow functional separation of bladder and urethral activity. A PE-50 catheter (Clay Adams) was inserted into the bladder through the top of the bladder wall to record intravesical pressure, and a PE-50 catheter (Clay Adams) was inserted from the urethra to record UPP. UPP test methods have been described in detail in our previous study 10. In brief, the bladder was infused with physiological saline at a rate of 0.04 mL/min to the threshold volume (0.4-1mL), which induced isovolumetric rhythmic contractions. The urethral catheter was continuously infused with physiological saline at a rate of 0.04 mL/min. PowerLab (AD Instruments) was used for data acquisition and manipulation. When the rhythmic bladder contractions stabilized for at least 30 min, the maximum amplitude of isovolumetric contractions and the intravesical pressure threshold for inducing urethral relaxation were measured. The following parameters were measured: UPP nadir, the lowest pressure during reflex urethral relaxation; Baseline UPP, mean pressure over 30 min without an increase in intravesical pressure induced by the micturition reflex. UPP change was calculated as baseline UPP minus UPP nadir.

### *In vitro* functional studies with organ bath test on the contractile force of the bladder and urethra

The contractile forces of the bladder and urethra muscle strips were performed by using organ bath (n=6 per group). Organ bath test methods have been described in detail in our previous study 11. In brief, rats were euthanized in a CO_2_ chamber followed by cervical dislocation. Bladder and urethra were dissected free as a block and immersed in a petri dish containing Krebs-Henseleit solution [containing (in mM) 117 NaCl, 4.7 KCl, 2.5 CaCl_2_, 1.2 MgSO4, 1.2 KH_2_PO_4_, 25 NaHCO_3_, and 11glucose]. Each strip was equilibrated unstretched for 30 min. A load of 1.0 g was applied to each strip by micrometer adjustment, and the load was readjusted to this level 30 min later. Changes in the tone of the strips were measured isometrically using force transducers, and the data were recorded using the Chart v3.6.9 software and the PowerLab/16sp data acquisition system (AD Instruments).

Electrical-field stimulation (EFS) was applied to the isolated balder and urethral rings placed between two platinum electrodes (1 mm diameter) connected to a Grass S88 stimulator (Astro-Med Industrial Park), respectively. Frequency- response curves (1-32 Hz) were elicited by stimulating the tissues for 10 s with pulse of 1 ms width at 50 V and 2 min intervals between stimulations. After tissues were washed three times, bladder muscle strips were used to examine the cumulative concentration-response curves to the contractile agonist Carbachol (1nM to 100 µM) stimulation. Contraction was produced using a single concentration of KCL (80 mM) in urethral muscle strips. Bladder and urethral contractions were completely abolished by prior incubation with a voltage-gated sodium channel blocker, tetrodotoxin (TTX, 1 µM), confirming the neurogenic nature of responses. Contraction response data were normalized to the wet weight of the respective urethral ring. Cumulative concentration response curves to carbachol in the bladder muscle strips were constructed, and contractile forces to 80 mM of KCL in the urethral muscle strips were also measured.

### Haematoxylin & Eosin and Masson's staining in bladder and urethra

The rats were killed with an overdose of pentobarbital (100 mg/kg, sigma, USA), and were transcardially perfused with 4% paraformaldehyde. The entire bladder and urethra were then removed. Transverse 8-μm stions of the collected segments were cut by a freezing microtome and mounted on slides. Every 10th stion from the bladder and urethra of each NC and DM rat were subsequently stained with haematoxylin and eosin (H&E) and Masson.

For H&E staining, the slides were immersed in hematoxylin at RT for 30 s, rinsed with running water until transparent, stained with eosin at RT for 30 s and then rinsed again with water. The slides were then air-dried at RT. Subsequently, the slides were sequentially immersed twice in 95% ethanol solution, twice in 100% ethanol, twice in a solution of 50% ethanol and 50% xylene and twice in 100% xylene. The slides were then observed under a light microscope (Olympus Corporation).

For Masson's staining, the staining process was as follows: (1) Deparaffinize stions if necessary and hydrate in distilled water. (2) Preheat Bouin's Fluid in a water bath to 56-64°C in a fume hood or very well ventilated area. (3) Place slide in preheated Bouin's Fluis for 60 minutes followed by a 10 minutes cooling period. (4) Rinse slide in tap water until stion is completely clear. (5) Rinse once in distilled water. (6) Mix equal parts of Weigert's (A) and Weigert's (B) and stain slide with working Weigert's Iron Hematoxylin for 5 minutes. (7) Rinse slide in running tap water for 2 minutes. (8) Apply Biebrich Scarlet / Acid Fuchsin Solution to slide for 15 minutes. (9) Rinse slide in distilled water. (10) Differentiate in Phosphomolybdic /Phosphotungstic Acid Solution for 10-15 minutes or until collagen is not red. (11) Without rinsing, apply Aniline Blue Solution to slide for 5-10 minutes. (12) Rinse slide in distilled water. (13) Apply Acetic Acid Solution (1%) to slide for 3-5 minutes. (14) Dehydrate very quickly in 2 changes of 95% Alcohol, followed by 2 changes of Absolute Alcohol. (15) Clear in Xylene or Xylene Substitute and mount in synthetic resin. Finally, the stions were sealed with neutral gum. Blue collagen fibers, red muscle fibers, red cellulose and red blood cells were observed under a microscope (Olympus Corporation).

### Statistics Analysis

All data are expressed using mean and standard error (mean ± SE). GraphPad Prism software (ver. 6.01, Inc., CA, USA) was used for statistical analysis. One-way or two-way ANOVA followed by Dunnett's multiple comparisons test were used, or Student's t-test was used to assess the results. A P-value < 0.05 was considered significant.

## Results

### Metabolic cage study

**Figure 1:**
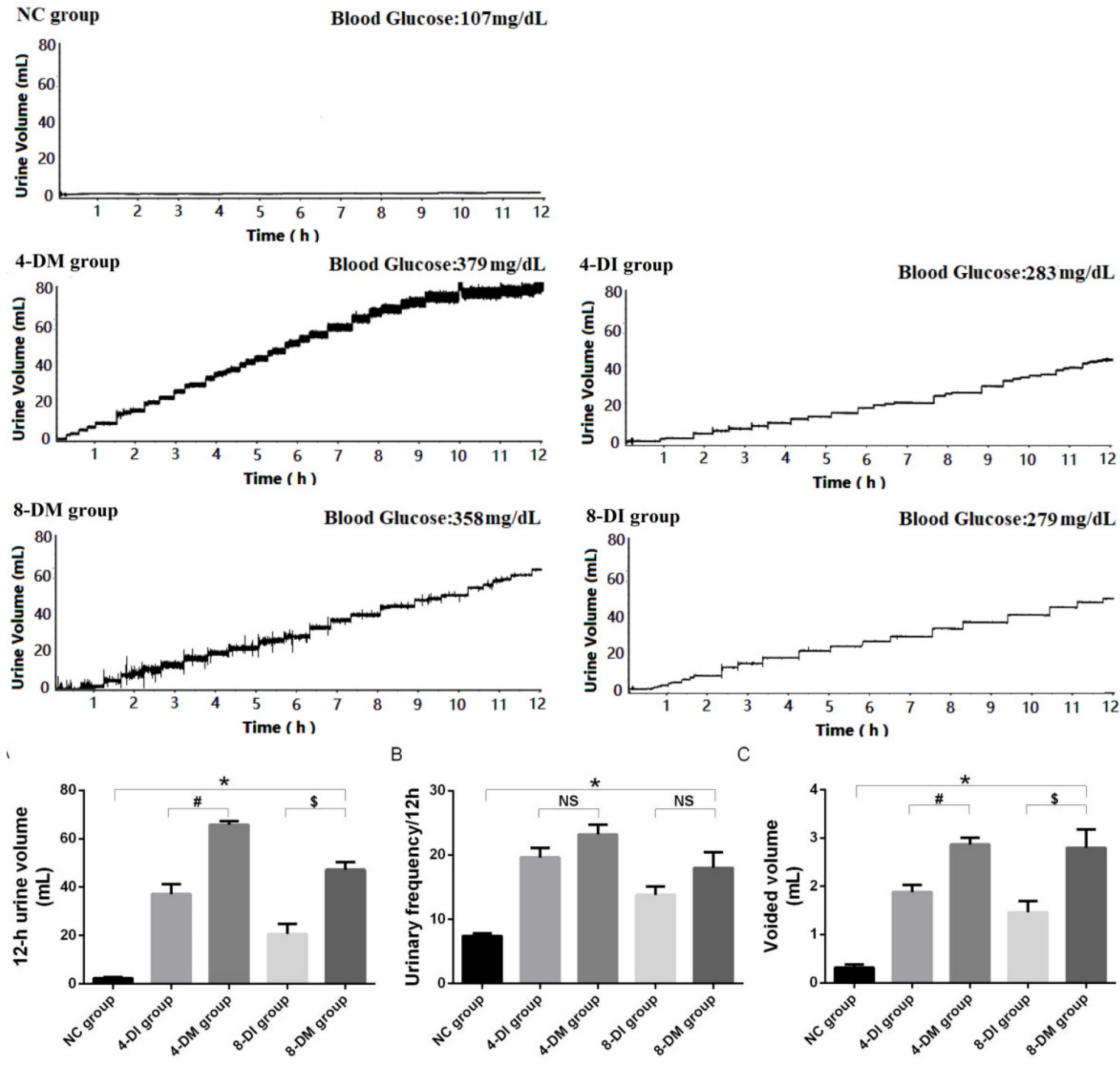
Voiding patterns in metabolic cage study. Diabetes mellitus (DM) and insulin-treated DM groups exhibited an increase in 12-h urine volume, urinary frequency/12 h and voided volume compared to normal control (NC) rats. The 4- and 8-weeks insulin-treated DM group showed the significant smaller values in 12-h urine volume and voided volume compared to the 4- and 8-weeks DM group, respectively. *P < 0.05 compared to the NC group, #P < 0.05 compared to the 4 weeks insulin-treated DM group, $ P < 0.05 compared to the 8 weeks insulin-treated DM group.

As is shown in Fig. 1, DM and DI groups exhibited a significant increase in 12-h urine volume, urinary frequency/12 hours and voided volume at each point. Moreover, 4 and 8-DM groups had a significant increase in 12-h urine volume and voided volume than 4 and 8-DI group, respectively. In normal control (NC), 4-DI, 4-DM, 8-DI and 8-DM groups of rats, urinary frequency/12 hours were 7.4 ± 0.9, 19.6 ± 3.4, 23.2 ± 3.4, 13.8 ± 3.0 and 18.0 ± 5.4, respectively. 12-h urine volume was 2.4 ± 1.2, 37.1 ± 9.3, 65.7 ± 3.7, 20.7 ±9.2 and 47.3 ± 7.2 (mL), respectively. VV was 0.3 ± 0.1, 1.9 ± 0.3, 2.9 ± 0.3 ,1.5 ± 0.5 and 2.8 ± 0.9 (mL), respectively.

### *In vivo* functional studies with cystometry and EUS-EMG with open urethra

**Figure 2:**
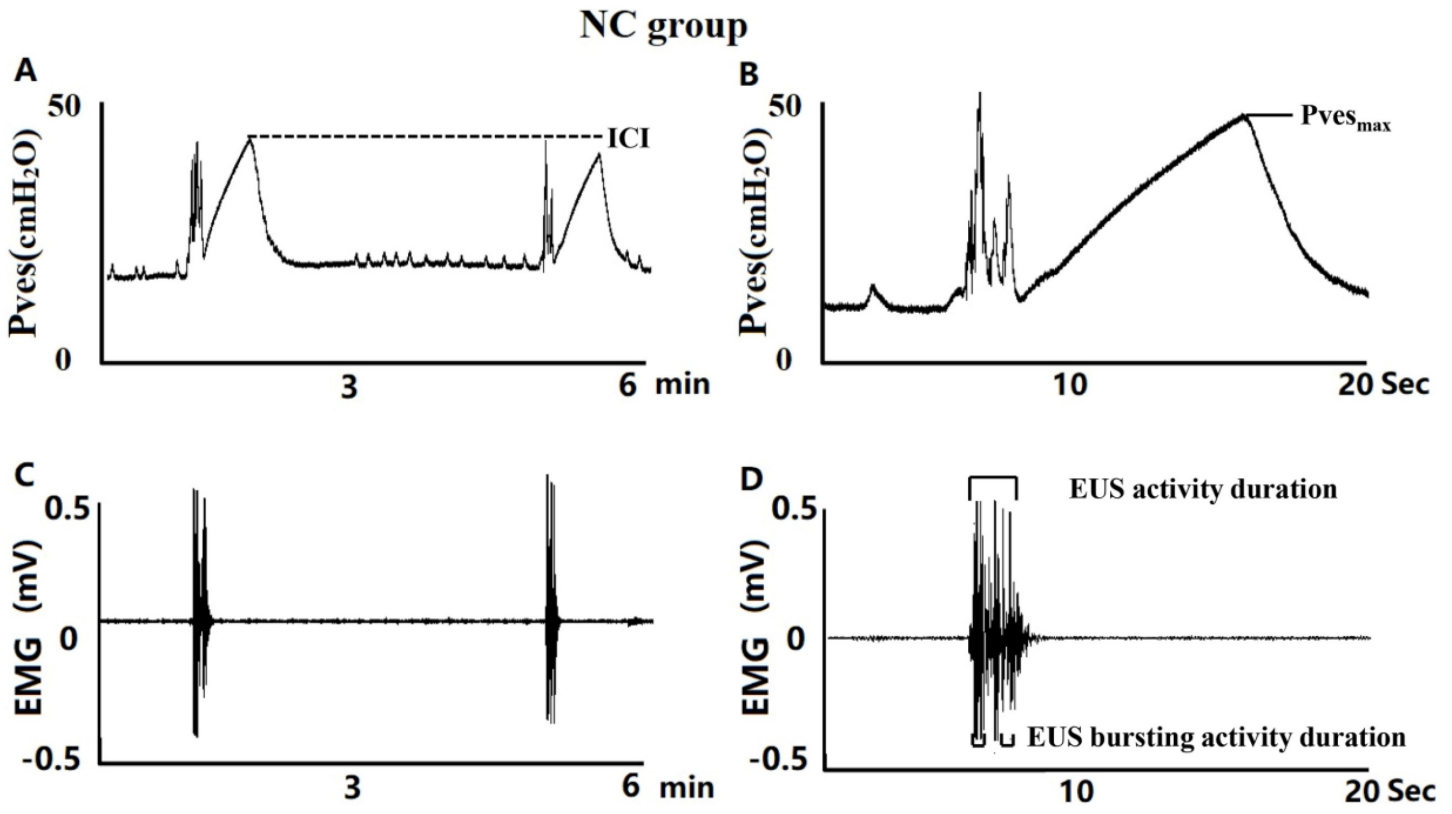
The typical pattern of the intravesical pressure (top trace) and EUS-EMG activity (bottom trace), recorded during a continuous transvesical infusion CMG measurement in a urethane-anesthetized normal control (NC) rat. B and D are traces at an expanded time scale of A and C, respectively. Tonic EUS-EMG activity preceded the large rise in intravesical pressure and shifted to a bursting pattern at the peak of the bladder contraction before the onset of voiding. ICI, intercontraction interval; Pves_max_, maximal intravesical pressure, is not identical with the micturition pressure, and the true micturition pressure is the pressure at which fluid starts to flow.

**Normal control rats (n=5).** Fig.2 shows typical CMG and EUS-EMG recordings acquired from urethane anaesthetized rats. In the NC group, bladder capacity was 0.58±0.04 ml, VV was 0.53±0.03 ml, VE was 91.05±1.73%, Pves_max_ was 40.78 ± 0.77 cmH_2_O, ICI was 5.84± 0.43 min, EUS activity duration was 4.28 ± 0.25 s and EUS activity bursting activity was 2.66 ± 0.16 s as shown in Fig. 2.

**Figure 3:**
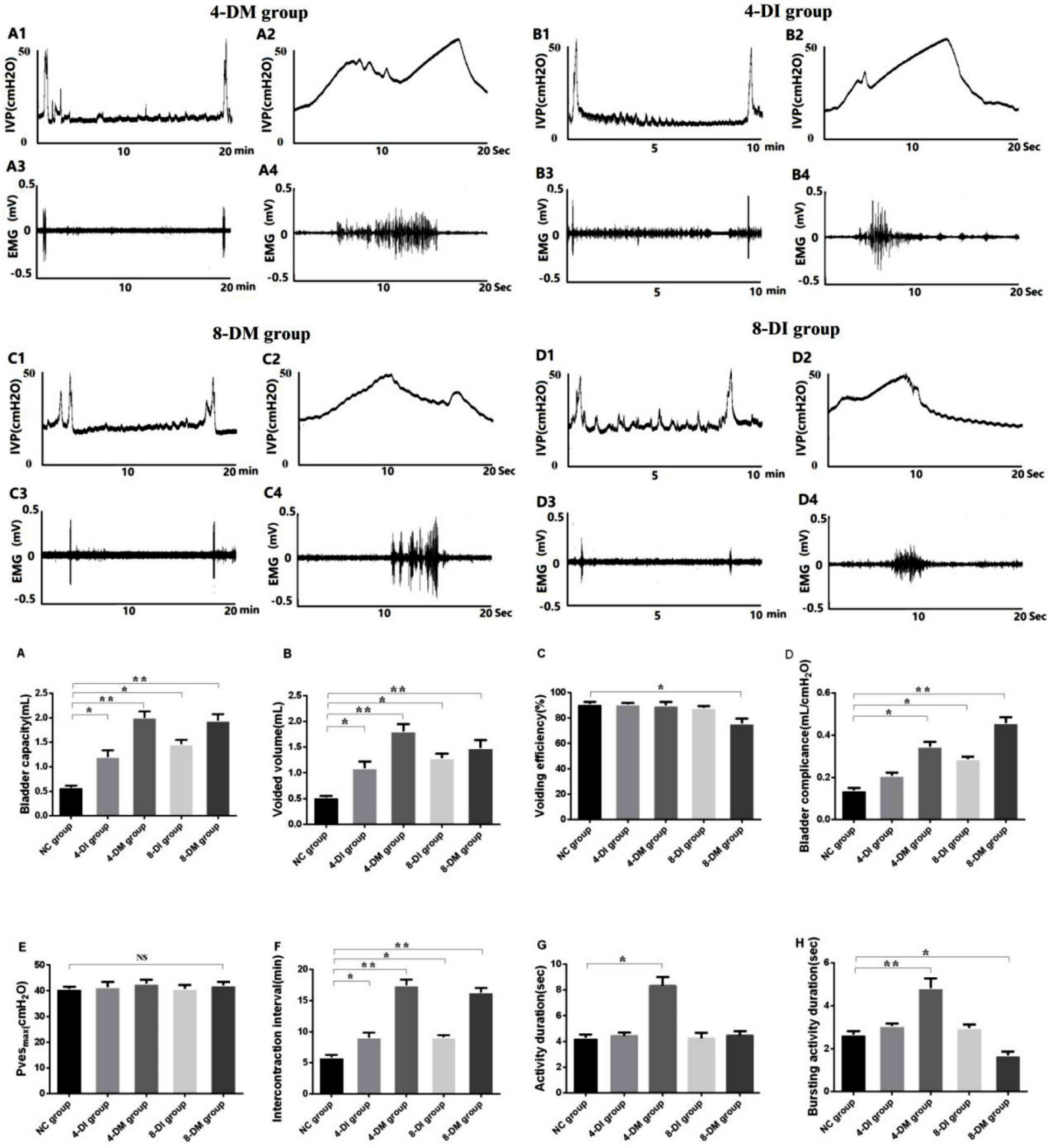
Representative traces showing CMG and EUS-EMG activity in urethane-anesthetized DM and insulin-treated rats. It is clear that ICI was increased in DM rats, and the EUS bursting activity duration was increased in 4 weeks DM rats, but decreased in 8weeks DM rats. Histograms show that bladder capacity and voided volume were significantly increased in DM and DI groups, whereas voiding efficiency was significantly decreased in the 8 weeks DM group. No significant difference in Pves_max_ was observed in all those groups. *P< 0.05, **P<0.01 vs. corresponding NC group.

**DM and insulin-treated DM animal models (n=5 per group).** As is shown in Fig. 3, typical CMG and EUS-EMG showed longer ICIs, EUS activity duration and EUS bursting activity duration in 4-DM group compared to the NC groups. Bladder capacity, VV and ICI have a significant increase in DM and DI groups; VE and EUS bursting activity duration in 8-DM group were significant lower compared to the NC group. However, no significant difference was observed in Pves_max_ among those groups. Additionally, bladder compliance was significant higher in 4 & 8-DM groups and the 8-DI group compared to the NC group.

### Changes in UPP parameters under isovolumetric conditions in DM and insulin-treated DM rats

**Figure 4:**
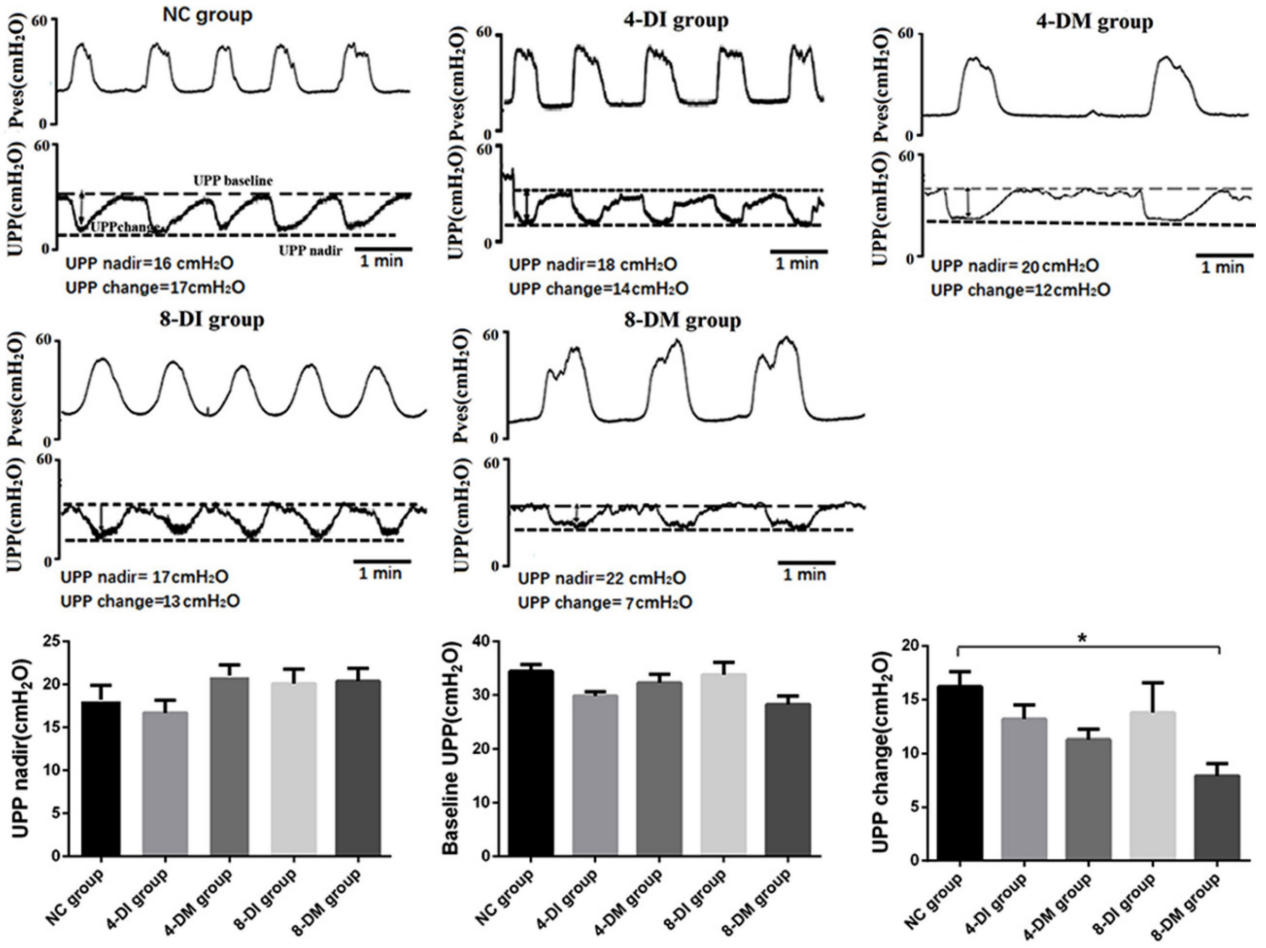
Representative traces showing various parameters in simultaneous recordings of intravesical pressure under the isovolumetric condition and UPP in urethane-anesthetized rats. Histograms of UPP changes shows an obviously decrease in the 8 weeks DM group (C). UPP, urethral perfusion pressure; UPP change, UPP baseline minus UPP nadir. *P< 0.05, vs. corresponding NC group.

As is shown in Fig. 4, in NC, 4-DI, 4-DM, 8-DI and 8-DM groups of rats, the value of UPP nadir were 18.20 ± 1.68, 16.66 ± 1.49, 21.01 ± 1.28, 20.07 ± 1.72, 20.36 ± 1.53 (cmH_2_O), respectively; the value of baseline UPP were 34.40 ± 1.36, 29.85 ± 0.33, 32.30 ± 1.60, 33.84 ± 2.25, 28.28 ±1.54 (cmH_2_O), respectively; the value of UPP change were 16.20 ± 1.40, 13.19 ± 1.30, 11.28 ± 0.94, 13.78 ± 2.79 and 7.93 ± 1.14 (cmH_2_O), respectively. Histograms showed that there was no significant difference in UPP nadir and baseline UPP among the groups (Fig. 4 A, B), whereas UPP changes in the 8-DM group were significantly lower than the NC group (Fig. 4 C), indicating that impaired urethral relaxation during voiding exists in the 8-DM group.

### *In vitro* functional studies with organ bath test on the contractile force of the bladder and urethra

**Figure 5:**
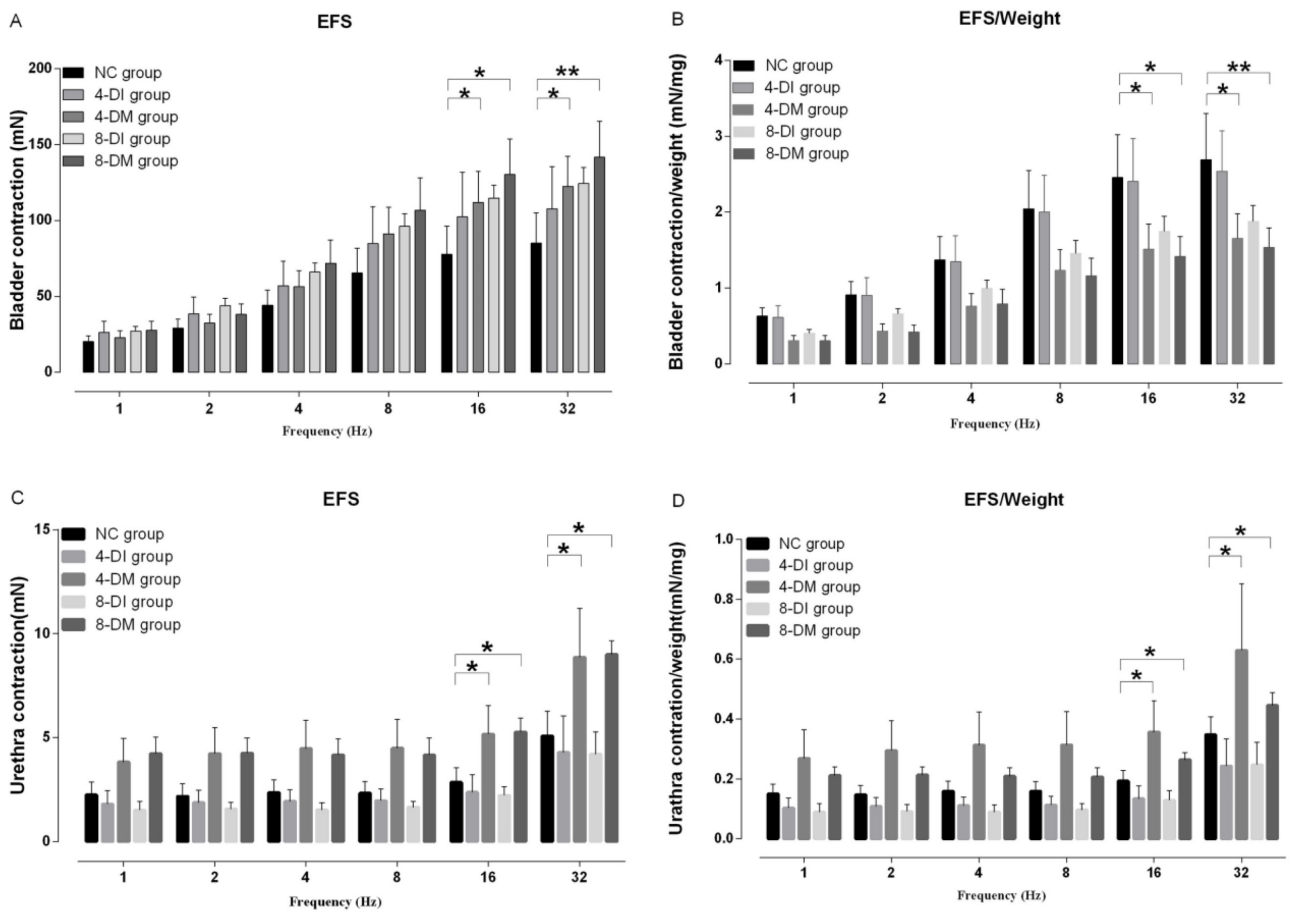
Histograms showing the contraction responses to electrical-field stimulation (EFS, 1-32 Hz) in the bladder (A, B) and urethra muscle strips (C, D) from normal control (NC), insulin-treated DM and DM groups. Data represent the mean ± S.E.M. (n = 6). *P< 0.05, **P<0.01 when compared to the NC group.

**Figure 6:**
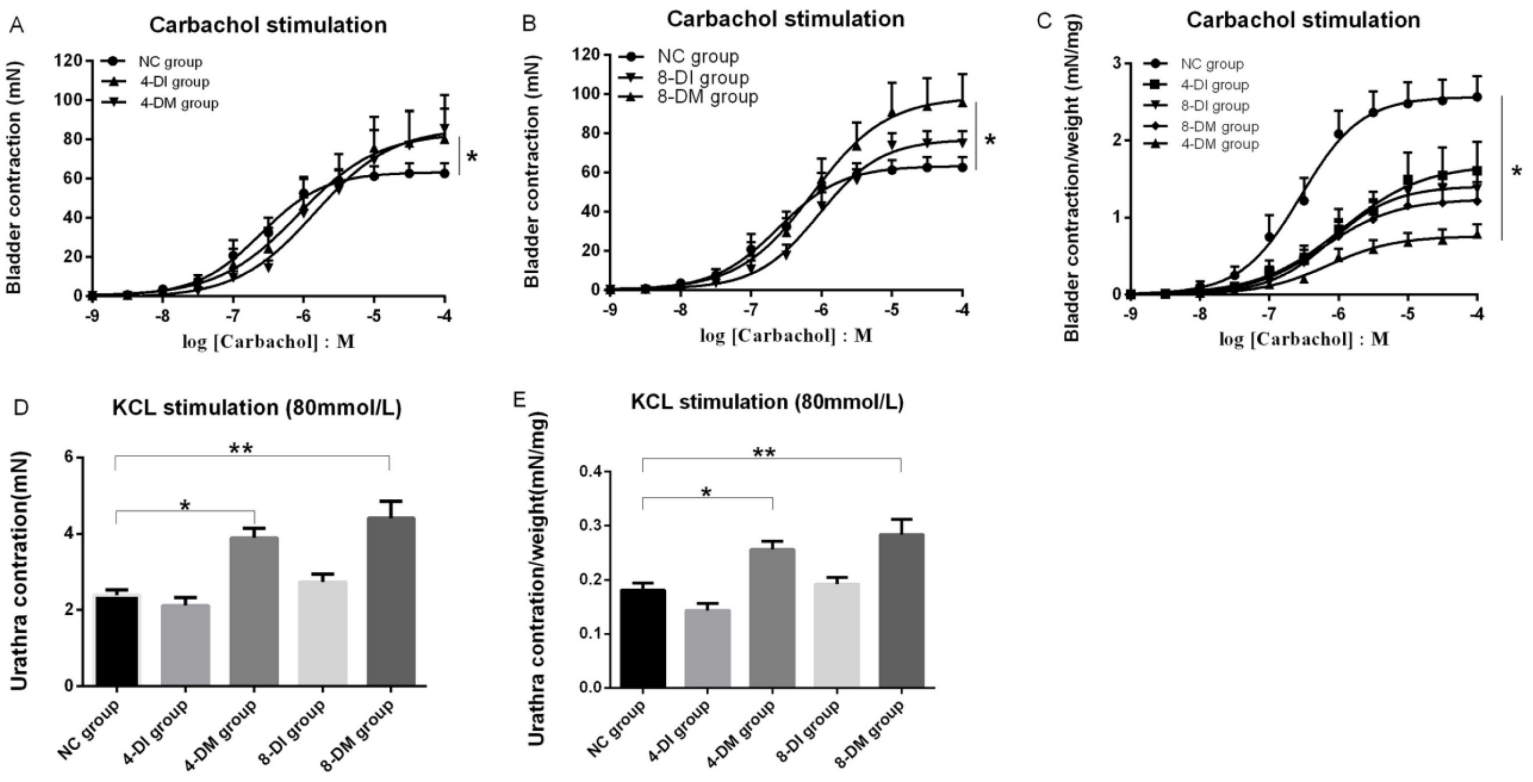
Cumulative concentration-response curves to the contractile agonist Carbachol (1nM to 100 µM) stimulation in bladder strips (A-C). Histograms show the urethral contraction in response to KCL (80mM) stimulation (D, E), *P< 0.05, **P<0.01 when compared to the NC group.

In a separated set of experiments, EFS (1-32Hz) induced frequency-dependent contractions in bladder strips from all the groups. After EFS stimulation, bladder contractions were significantly higher in 4 and 8-DM groups compared to the NC group at the frequencies of 16 and 32Hz (Fig. 5A). However, maximum bladder muscle contraction per gram of the tissue to EFS stimulation was significantly lower in 4 and 8-DM group compared to the NC group at the frequencies of 16 and 32 Hz (Fig. 5 B). Moreover, concentration-response curves were generated to assess the contraction profile in response to muscarinic receptor activation using carbachol (1nM-100 µM) across the different groups. Fig. 6 shows that the bladder contractions to carbachol stimulation in 4 and 8-DM groups were larger than 4 & 8-DI and NC groups (Fig. 6A, B). However, the contractions per milligram of bladder smooth muscle to carbachol were significantly decreased in 4 and 8-DM groups compared to the NC group (Fig. 6 C).

After EFS (1-32HZ) stimulation, the maximum urethral contraction and maximum urethral contraction per gram of the tissue to EFS stimulation were increased in 4 and 8-DM groups compared to the NC group, especially at the frequency of 16 and 32 Hz (Fig. 5C, D). Additionally, maximum urethral contraction and to maximum urethral contraction per gram of the tissue to KCL stimulation were significantly increased in 4 and 8-DM group compared to the NC group (Fig. 6D, E).

### H&E and Masson's staining

**Figure 7:**
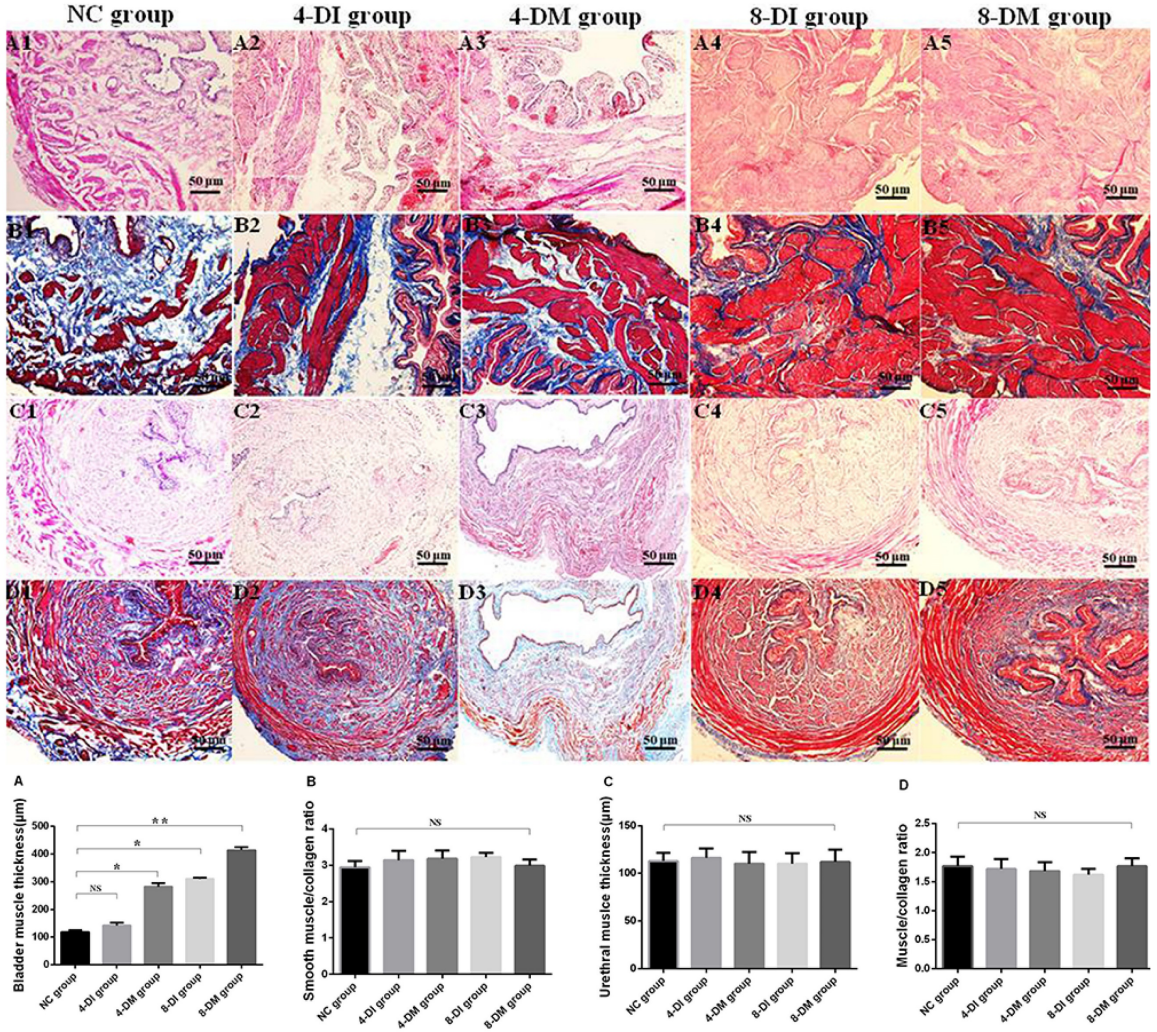
Representative photomicrographs of haematoxylin and eosin (H&E) and Masson's staining (muscle: red) of bladder in normal control (NC), diabetes mellitus (DM) and insulin-treated DM rats (n=5 per group). (A1-A5) and (C1-C5) were original magnification using H&E staining (40x). (B1-B5) and (D1-D5) were original magnification (40 x) with Masson's staining. Histograms show the hypertrophic changes in the bladder detrusor muscles in DM rats, whereas urethral muscle layer thickness showed no significant difference in DM groups compared to the NC rat. *P < 0.05 vs corresponding NC group. Scale bar, 50μm.

H&E and Masson's staining showed that DM caused hypertrophic changes in the bladder detrusor muscle with the time-dependent progress in DI and DM groups. Histogram data revealed that the bladder muscle thickness was significantly increased compared to the NC groups in 4 & 8-DM groups and the 8-DI group (Fig. 7A), whereas the ratio of muscle and collagen was not significantly changed in any of the groups (Fig. 7B). Additionally, no significant difference of the urethral muscle thickness and the ratio of muscle and collagen were observed among the groups (Fig. 7C, D).

## Discussions

Diabetes mellitus is a metabolic disorder caused by an absolute or relative deficiency of insulin, which is a debilitating and costly disease with multiple serious complications. Lower urinary tract dysfunction (LUTD) is among the most common complications of DM 15. Although STZ-induced DM rats have been widely used as a rodent model for LUTD, this is an acutely-induced, severe DM model with high glucose levels that can easily damage other systems in a short period, often leading to early animal death. Hence, this STZ-induced DM model may not be suitable for studying the pathophysiological condition encountered in DM patients, especially those with type 2 DM that is induced by insufficient insulin production and insulin resistance. DM causes the increases in drinking and voiding volume in the early stage, which may be due to the hyperglycemia-induced osmotic polyuria 12. As the course of the disease progressed, bladder and urethral function could be damaged by DM, leading to LUTD. Our and other studies have shown the time-dependent alteration of bladder and urethral function in diabetic rats 7, 12, whereas time-dependent changes of LUT function in low-dose insulin-treated DM were not established in previous studies. The current study confirms that diabetic rats have altered micturition patterns, which varied at different time points, including alterations in voided volume, micturition episodes/12 hours and voided volume per micturition (Fig.1). DM and DI groups exhibited a significant increase in 12-h urine volume, urinary frequency/12 hours and voided volume at each point, and 4 and 8-DM group had a significant increase in 12-h urine volume and VV compared to 4 and 8-DI group, respectively. These results demonstrate that the low-dose insulin-treated DM rat is a stable animal model, which can be reliably used for the long-term study of DM-induced bladder and urethral dysfunction.

DM-induced bladder dysfunction (DBD) has been widely reported in STZ-induced animal model. The progress of DBD has been defined as two states, compensatory and decompensatory bladder. The compensatory state that occurs soon after the onset of diabetes, characterized by overactive detrusor, enhanced contractility, and bladder hypertrophy 12. Subsequently, decompensation occurs in the late stage of diabetes, characterized by slow urine flow, decreased maximum voiding pressure, and symptoms of urine retention and dysuria 13. The finding of the current study shows that DM could increase the bladder capacity, VV and inter-contraction interval as early as 4 weeks, and this effect becomes more prominent at 8 weeks (Fig.3A, B, F). However, a significant decrease in voiding efficiency was observed only in 8 weeks DM (Fig.3C). Moreover, we also observed that ICI and bladder compliance were gradually increased in DI and DM group as time progressed but no significant change was observed in 4 weeks DI group (Fig.3D), suggesting low-dose insulin treatment may slow the emergence of bladder dysfunction induced by diuresis-induced bladder overdistention, in line with our previous study, in which, however, only 8 weeks DM rats were used to assess the DM-induced DBD without EUS-EMG recordings 14.

Although DBD has been widely reported in previous studies, little is known about the time-dependent changes in DM-induced urethral dysfunction. Because the urethra consists of the external urethral sphincter (EUS) and the smooth muscle sphincter, DM-induced urethral dysfunction is mainly characterized by impaired relaxation of the EUS and smooth muscles. In normal rats, EUS activity can be divided into tonic activity and bursting activity. Tonic activity of the EUS reflects a closure phase of the urethral outlet during urine storage, while bursting activity reflects rhythmic opening and closing of the outlet to produce a pulsatile flow of urine, which is commonly observed in rodents 15. It is assumed that the DM-induced EUS dysfunction would increase outlet resistance and, consequently, decrease voiding efficiency. In our present study, we observed that EUS bursting activity duration was significantly increased in 4-DM group, whereas deceased in 8-DM group, which may suggest that DM-induced EUS dysfunction may be varied from overactivity to underactivity as time progress. Moreover, there were no significant changes of EUS bursting activity in DI groups, indicating that low-dose insulin may slow the progress and protect against the EUS dysfunction.

Furthermore, *in vivo* functional study with UPP recordings, the results found that UPP change significantly decreased in the 8-DM group, which suggested that DM could induce urethral relaxation dysfunction after 8 weeks DM, whereas low-dose insulin could protect against impaired urethral relaxation during voiding. In *in vitro* organ bath study, we found that bladder smooth muscle strips contractions normalized against tissue weight in response to EFS and carbachol stimulation have significantly decreased in DM groups, but not in DI and normal rats. These results suggest that M_2_ and M_3_ muscarinic receptors are possibly downregulated in the urothelium of the STZ-induced diabetic rats as activation of M_2_ and M_3_ receptors in the urothelium reportedly increases sensory nerve activity to facilitate the detrusor contraction 16. Also, we also examine urethra contractions to EFS and KCL stimulation. Urethral smooth muscle strips contractions normalized against tissue weight in response to EFS and KCL stimulation have been increased in DM groups, but not in DI vs. normal rats. These results may be ascribed to the upregulation of α_1_-adrenergic receptors in the urethral smooth muscle of STZ-induced diabetic rats 17, 18. Also, an increase of ATP-sensitive potassium channel in the urothelium also can increase sensory nerve activity to facilitate urethral muscle contraction 19. Furthermore, the results from H&E and Masson's staining revealed a progressive increase in the thickness of the bladder smooth muscle layer in 8-DI and 4 & 8-DM groups over time. However, the thickness of the urethral muscle layer did not show significant changes among the groups. Taken together, it is likely that DM-induced LUTD is time-dependent and include decreased bladder contractility evident as reduced muscle strip contraction per tissue weight (4 & 8-DM rats) and reduced urethral relaxation evident as lower UPP reduction (8-DM rats) and enhanced urethral contraction upon KCL or EFS stimulation (4 & 8-DM rats). Also, the results of this study indicate that DM induces bladder structural remodeling that can be delayed by low-dose insulin treatment as the bladder wall thickness was found in both 4 and 8-DM rats, but only in 8-DI rats, possibly due to a protective effect against osmotic polyuria.

Based on the above findings, the use of sustained-release low-dose insulin injections can create a relatively stable model of DM-induced LUTD. Moreover, low-dose insulin demonstrates a certain protective effect against lower urinary tract dysfunction, potentially mediated through its interaction with insulin receptors in the lower urinary tract 20. However, it is important to note that low-dose insulin cannot fully reverse the damage caused by diabetes. This may be attributed to other underlying mechanisms, as the pathophysiology of lower urinary tract dysfunction in diabetes involves not only osmotic diuresis but also oxidative stress, inflammation, and other mechanisms 21. Further research is needed to explore these mechanisms in depth.

There are some limitations in this study. Firstly, we did not use neuromuscular blocking agents such as α-bungarotoxin to separate the smooth and striated muscle activity of the urethra, and therefore EFS-induced transient contraction in urethral ring tissues may be related to EUS contractions. Secondly, UPP recordings were performed under urethane anesthesia, which might not reflect true functionality in the conscious condition. Thirdly, we did not assess the longer effects of the insulin treatment in DM and molecular mechanisms underlying DM-induced changes in the bladder and urethra. Thus, further studies will be planned to clarify these points in the future.

## Conclusions

DM-induced LUTD is characterized by the time-dependent functional and structural remodeling in the bladder and urethra, which shows the hypertrophy of the bladder smooth muscle, impaired urethral smooth muscle relaxation and EUS dysfunction. Low-dose insulin can protect against diuresis-induced bladder overdistention and preserve urethral relaxation; however but the longer effects of DM with or without low-dose insulin and molecular mechanisms underlying DM-induced LUTD should be further explored.
